# Supplementary material for: Predicting Region of Interest in Human Visual Search Based on Statistical Texture and Gabor Features
Source: ArXiv. 2026 Jan 12:arXiv:2601.07998v1. Preprint. [Version 1] (PMC12869375)
Supplement: Supplement 1 [file NIHPP2601.07998v1-supplement-1.pdf]

6 Supplements Material

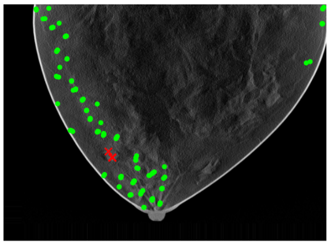

Figure 1: "Scattered" DBT pipeline A result

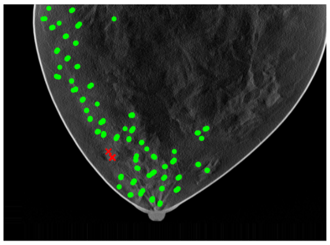

Figure 2: "Scattered" DBT pipeline B result

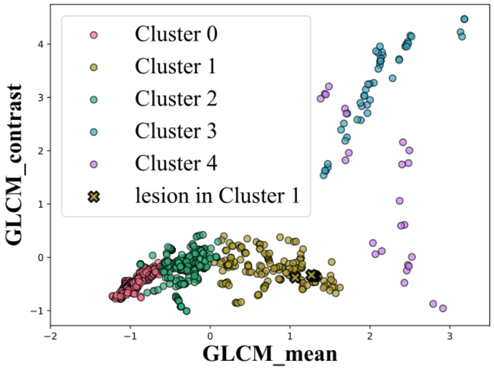

Figure 3: "Scattered" density DBT scattered plot

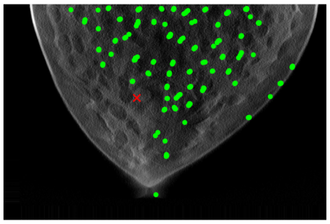

Figure 4: "Heterogeneously dense" DBT pipeline A result

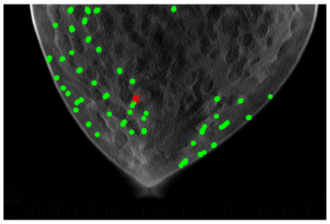

Figure 5: "Heterogeneously dense" DBT pipeline B result

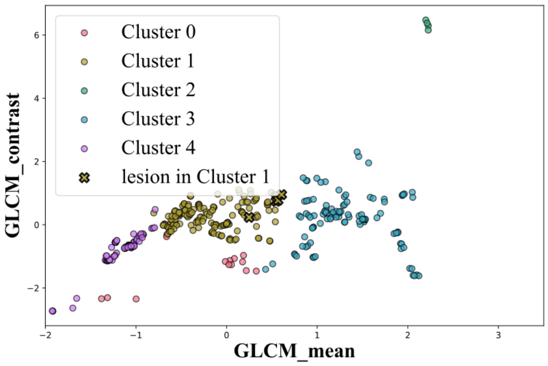

Figure 6: "Heterogeneously dense" density DBT scattered plot
